# Structure and Properties of Octenyl Succinic Anhydride-Modified High-Amylose Japonica Rice Starches

**DOI:** 10.3390/polym13081325

**Published:** 2021-04-18

**Authors:** Wei Zhang, Bei Cheng, Jiahui Li, Zaixi Shu, Pingping Wang, Xuefeng Zeng

**Affiliations:** 1 College of Food Science and Engineering, Wuhan Polytechnic University, Wuhan 430023, China; tm13cheng@163.com (B.C.); lijiahui970805@163.com (J.L.); shuzaixi@163.com (Z.S.); wpp1984225@163.com (P.W.); 2 Key Laboratory for Deep Processing of Major Grain and Oil (Wuhan Polytechnic University), Ministry of Education, Wuhan 430023, China; 3 School of Liquor and Food Engineering, Guizhou University, Guiyang 550000, China; heiniuzxf@126.com

**Keywords:** rice starch, octenyl succinic anhydride, emulsion stability, digestibility

## Abstract

Starches rich in amylose are promising functional ingredients for calory-reduced foods. In this research, a high-amylose Japonica rice starch (amylose content 33.3%) was esterified with octenyl succinic anhydride (OSA) to improve the functional properties. The OSA-modified derivatives were evaluated for structure and functional properties, with OSA-modified normal Japonica rice starch (amylose content 18.8%) used as control. Fourier transform infrared spectra confirmed the introduction of OSA groups to starch. OSA modification made little change to morphology and particle size of high-amylose starch, but decreased the relative crystallinity and pasting temperature and increased the pasting viscosity, swelling power, emulsifying stability, and resistant starch (RS) content. The changes of properties were related to the degree of substitution (DS). Typically, OSA-modified high-amylose starch at DS of 0.0285 shows polyhedral-shape granules, with a volume-average particle diameter of 8.87 μm, peak viscosity of 5730 cp, and RS content of 35.45%. OSA-modified high-amylose starch had greater peak viscosity and RS content and lower swelling power than OSA-modified normal starch of similar DS, but the two kinds of derivatives did not have a significant difference in emulsifying stability. The OSA-modified high-amylose Japonica rice starch could be used as an emulsifier, thickener, and fat replacer in food systems.

## 1. Introduction

Native starches often perform poorly in industrial applications and are tailored through physical or chemical modification to develop desirable functional properties, such as pasting properties, dispersity, and digestibility [1,2]. Octenyl succinic anhydride (OSA) is a commonly used esterification agent for modifying starches. OSA modification incorporates hydrophobic groups onto starch molecules, and the derivative is amphiphilic, which can act as an emulsion stabilizer [3,4]. The OSA-modified starch has been approved as an additive in the USA since 1972. Because of its favorable characteristics, the consumption of OSA-modified starch has been growing rapidly worldwide.

The properties of OSA-modified starch have been affected by many factors, including starch type, amylose content, degree of substitution (DS), etc. Wen et al. [5] found that OSA modification decreased the gel hardness of oat, rice, and quinoa starches, but increased that of waxy maize and amaranth starches. Lopez-Silva et al. [6] reported that OSA treatment reduced the extent of starch digestibility, and the amylose content was positively linked to resistant starch content of the derivative. Song et al. [7] found that amylose content was negatively correlated with the peak viscosity and breakdown viscosity of OSA-modified indica starch. Yu et al. [8] found that emulsions stabilized with OSA-modified starch exhibited increased stability as DS increased.

Rice (*Oryza sativa* L.) is one of the most important cereal crops, providing more than half of the world’s staple food. The Asian cultivated rice grown worldwide is classified into two major subspecies, Indica and Japonica. Indica rice is the major type of rice grown in the tropics and subtropics, for example, central and southern China, India, Pakistan, and Indonesia [9,10]. Japonica rice is mainly distributed in cooler zones of the subtropics and in the temperate zones, for example, northeast China, Korea, and Japan [11,12]. Rice starch consists of tiny granules with a narrow size distribution. In previous research, OSA-modified indica rice starches have been widely investigated in structure and functional properties, and many applications have been developed, for example, stabilizing emulsions and substituting fat in foods [13,14,15,16]. To date, there are only limited studies available in this research area of OSA-modified Japonica rice starch [17].

Generally, the amylose content of Japonica rice starch is in the range of 0–20%. In recent years, considerable efforts have been paid to breeding techniques to modify the amylose content of Japonica rice starch. In this research, a high-amylose Japonica rice starch (amylose content 33.3%), a promising ingredient for calory-reduced food, was chemically modified with OSA. The structure and properties of the OSA-modified high-amylose Japonica rice starch were investigated and compared with OSA-modified normal Japonica rice starch (amylose content 18.8%). This research will be helpful for the design of modified starch with new and enhanced properties.

## 2. Materials and Methods

### 2.1. Materials

High-amylose Japonica rice (variety Jiangtangdao1) was kindly supplied by the Shanghai Academy of Agricultural Sciences (Shanghai, China). Normal Japonica rice (varieties of huai5) was obtained from a local grain trader (Hubei, China). Soybean oil (Golden Dragon Fish) was supplied by Yihai Kerry Group (Shanghai, China). Porcine pancreatic-amylase (P7545) and amyloglucosidase (A7095) were purchase from Sigma Chemical Co. (St. Louis, MO, USA). A glucose oxidase/peroxidase (GOPOD) kit was purchased from Megazyme International Ireland Ltd. (Bray Co., Wicklow, Ireland). Other chemical reagents were of analytical grade unless otherwise stated.

### 2.2. Isolation of Starch

Starch was isolated from white rice according to the method of Wang et al. [18]. According to the method of Lin et al. [19], the amylose contents of normal and high-amylose starches were determined as 18.8% and 33.3%, respectively. These two starches were coded as NormalST and HighAmyST, respectively. According to the method of Shi et al. [20], for NormalST, the A, B1, B2, and B3 chains of amylopectin, corresponding to the degree of depolymerization (DP) ranges of 6–12, 13–24, 25–36, and ≥37, were determined as 31.2%, 47.7%, 10.7%, and 10.3%, respectively; for HighAmyST, the four fractions were significantly different at 19.4%, 49.9%, 13.7%, and 17.1%, respectively.

### 2.3. Preparation of OSA-Modified Starch

Starch (100 g, dry weight) was dispersed in water (140 mL) with stirring. The pH of the slurry was adjusted to 8.5 with 1 mol/L NaOH at 25 °C. OSA (3%, 5%, or 8%, based on starch weight) was added to the slurry. The mixture was continuously stirred for reaction, with pH maintained at 8.5. After 6 h of reaction, the starch slurry was neutralized to pH 7.0 with 1 mol/L HCl and then centrifuged at 3000 r/min for 15 min (FC5706 Centrifuge, Ohaus, NJ, USA). The residue was washed three times with water and once with acetone, and then dried at 40 °C for 24 h. The degree of substitution (DS) of OSA-modified starch was determined by titration following the method described by Song et al. [21].

### 2.4. Fourier Transform Infrared Spectroscopy (FTIR) Analysis

The Fourier transform infrared (FTIR) spectra of native and OSA-modified starch samples were collected on a Nicolet Nexus 670 FTIR spectrophotometer (Thermo Scientific, Waltham, MA, USA). Starch samples were mixed with dried KBr powder in a ratio of 1:100 (*w*/*w*) and pressed into transparent tablets under infrared light before measurement. Measurements were made in the region 4000–400 cm^−1^ at a resolution of 4 cm^−1^ with a total of 64 scans per spectrum.

### 2.5. Scanning Electron Microscopy (SEM)

The morphologies of native and OSA-modified starch samples were examined and photographed using a scanning electron microscope (S-3000N, Hitachi High-Technologies Corp., Tokyo, Japan) at an accelerating voltage of 15 kV. Before examination, starch samples were sprinkled onto double-sided tape attached to a specimen holder and coated with gold in a vacuum evaporator.

### 2.6. Determination of Particle Size Distribution

The particle size distributions of native and OSA-modified starch samples were measured with the laser light scattering method, by using a Malvern Mastersizer 3000 instrument (Malvern Instruments Ltd., Worcestershire, UK). Distilled water was used as the suspension medium. Starch samples (100 mg) were dispersed in 5 mL of water and vortexed for 2 min (IKA Lab Dancer, Sigma-Aldrich Inc., St. Louis, MO, USA). The dispersion was then swiftly transferred into the sample cell of the particle size analyzer.

### 2.7. X-ray Diffraction (XRD) Analysis

The crystalline structures of native and OSA-modified starch samples were evaluated using an Xpert PRO diffractometer (PANalytical B.V., Almelo, the Netherlands), operating at 40 kW and 40 mA with a Cu Kα radiation source (λ = 0.154 nm). Starch samples were equilibrated to a moisture content of about 10% before the analysis. XRD patterns were acquired for a diffraction angle (2θ) range from 3° to 40° at a scanning speed of 10° min^−1^ and a scanning step of 0.033°. The relative crystallinity (%) was quantitatively estimated by using the Origin software (Version 8.0, Microcal Inc., Northampton, MA, USA) according to the method of Wang et al. [22].

### 2.8. Pasting Property Measurement

The pasting properties of native and OSA-modified starch samples were measured using a Rapid Visco-analyzer (RVA, Newport Scientific, Warriewood, New South Wales, Australia) according to the method of Chen et al. [23]. Starch samples (3.0 g, dry weight) were weighed exactly into an RVA canister, and distilled water was added to make a total weight of 28 g. The suspensions were equilibrated at 50 °C for 1 min, heated to 95 °C at a rate of 12 °C/min, held at 95 °C for 2.5 min, cooled to 50 °C at a rate of 12 °C/min, and held at 50 °C for 1.5 min. The speed of the mixing paddle was 960 rpm for the first 10 s and then 160 rpm for the remainder of the experiment. RVA parameters, such as pasting temperature (PT), peak viscosity time (Ptime), peak viscosity (PV), trough viscosity (TRV), final viscosity (FV), breakdown viscosity (BDV), and setback viscosity (SBV) were recorded using the Thermocline software provided with the instrument.

### 2.9. Swelling Power Determination

Swelling power of native and OSA-modified starches were determined by heating starch–water slurries in a water bath at temperatures ranging from 50 to 80 °C in 10 °C intervals, according to the procedures of Wang and Copeland [24] with some minor modifications. Briefly stated, the 4% starch slurry in deionized water was heated for 30 min at a specific temperature. During heating, the samples were swirled by hand after 10 and 20 min to resuspend precipitated starch. After heating, the slurries were cooled at room temperature for 10 min and then centrifuged at 4000 r/m for 20 min (Sorvall RC6 plus centrifuge, Thermo Scientific, Langenselbold, Germany), and the supernatant was removed and dried by evaporation at 105 °C. The sedimented swollen granules were weighed to determine swelling power (*g*/*g*) using the following formula:

Swelling power (*g*/*g*) = (weight of sedimented swollen granules)/(dry weight of original starch)

### 2.10. Emulsifying Stability Measurements

The emulsifying stability of native and OSA-modified starches was measured according to the method of Lin et al. [25] with a slight modification. Starch samples (12 g, dry weight) were suspended in water (78 g) and heated in a boiling water bath, with stirring for 20 min to ensure complete dispersion. Soybean oil (10 g) was blended with the OSA-modified starch solution, and the mixture was homogenized using a Nano DeBEE homogenizer (B.E.E. International Inc., South Easton, MA, USA) at 1000 bar for 10 cycles. Immediately after preparation, the emulsions (10 g) were poured into centrifuge tubes and were stored at 25 °C for 48 h. The emulsions were centrifuged at 3000 r/min for 20 min (FC5706 Centrifuge, Ohaus, NJ, USA) to determine the creaming indexes of fresh and stored emulsions according to the equation:

$$Creaming\;index\;\left(\%\right)=\frac{H_{s}}{H_{E}}\times 100$$

where *H_S_* is the height of the serum layer, and *H_E_* is the total height of the emulsion. A lower creaming index value indicates higher emulsion stability.

### 2.11. In Vitro Digestibility Assay

In vitro digestibility of native and OSA-modified starches was analyzed according to the method of Ma et al. [26]. Briefly, samples were suspended in a sodium acetate buffer, and the mixture was heated at 95 °C for 30 min. The gelatinized samples were treated with an enzyme mixture (pancreas α-amylase and amyloglucosidase) at 37 °C. The glucose content of the enzymatic hydrolysates at 0, 20, and 120 min of hydrolysis was determined to calculate the rapidly digestible starch (RDS, digested within 20 min), slowly digestible starch (SDS, digested between 20 and 120 min), and resistant starch (RS, undigestible starch after 120 min).

### 2.12. Statistical Analysis

Data are averages of duplicate observations and expressed as means ± standard deviations. An analysis of variance with a significance level of 5% was conducted, and Duncan’s test was applied to determine differences between means using the commercial statistical package (SPSS, Inc, Chicago, IL, USA).

## 3. Results and Discussion

### 3.1. Degrees of Substitution (DS) of OSA-Modified Japonica Rice Starches

**Figure 1:**
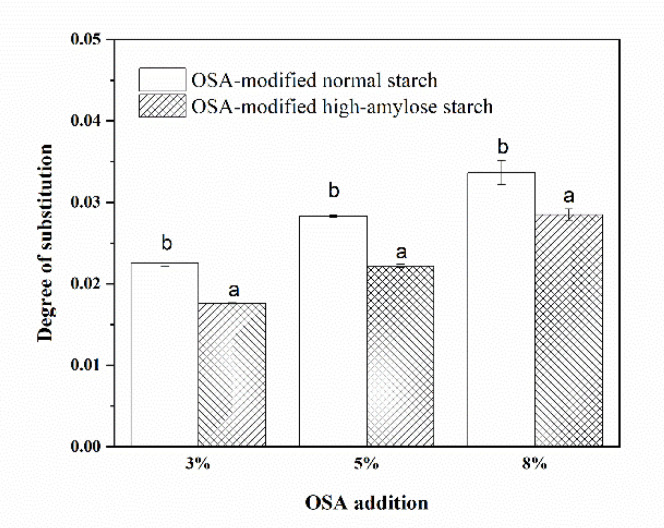
Degrees of substitution of normal and high-amylose Japonica rice starches after octenyl succinic anhydride (OSA) modification. At a specific OSA addition, bars with the same letter were not significantly different (α = 0.05).

The DS of OSA-modified normal and high-amylose Japonica rice starches as a response to OSA addition are shown in Figure 1. For both Japonica rice starch varieties, the DS of OSA-modified starches increased with increasing addition of OSA. At all three OSA additions, the DS of OSA-modified normal starches were higher than those of OSA-modified high-amylose starch. These results were consistent with No et al. [17], who reported that the waxy Japonica rice (amylose content 0.24%) had higher octenylsuccinylation activity than the normal Japonica rice starch (amylose content 16.69%). However, in the case of Indica rice starch, the amylose content showed a positive relation to OSA modification [13]. These results suggest that the DS of OSA-modified rice starch was influenced by OSA level, rice cultivar, and amylose content. The DS of OSA-modified normal starch at 5% OSA was 0.0283, which was coded as OSA-NormalST. The DS of OSA-modified high-amylose starch at 3% and 8% OSA were 0.0177 and 0.0285, respectively, which were coded as OSA-HighAmyST-1 and OSA-HighAmyST-2, respectively. OSA-NormalST and OSA-HighAmyST-2 did not have significant differences in DS.

### 3.2. Chemical Structures of OSA-Modified Japonica Rice Starches Determined by FTIR Spectroscopy

**Figure 2:**
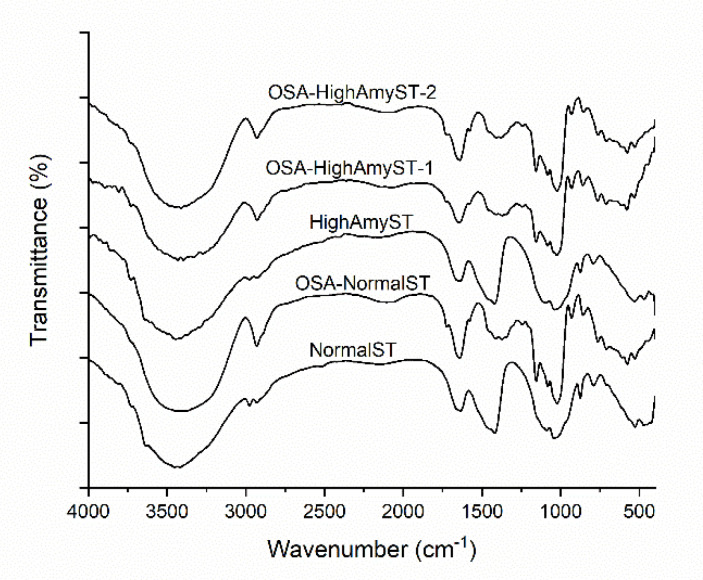
Fourier Transform Infrared (FTIR) spectra of normal and high-amylose Japonica rice starches with and without OSA modification. NormalST: native normal starch; OSA-NormalST: OSA-modified normal starch (DS = 0.0283); HighAmyST: native high-amylose starch; OSAHighAmyST-1: OSA-modified high-amylose starch (DS = 0.0177); OSA-HighAmyST2: OSA-modified high-amylose starch (DS = 0.0285).

The FTIR spectra of normal and high-amylose Japonica rice starches with and without OSA modification are shown in Figure 2. In the spectrum of native normal and high-amylose starches, the broad band appearing at 3460 cm^−1^ could be attributed to the vibration of O–H groups. The band at 2942 cm^−1^ was due to C–H stretching related with ring methane hydrogen atoms. The characteristic peak at 1643 cm^−1^ originated from the tightly bound water present in the starch. The peaks at 800–1200 cm^−1^ were due to stretching of the C–O bond.

For both normal and high-amylose starches, a new peak at 1571 cm^−1^ emerged after OSA modification. This peak was ascribed to the asymmetric stretching vibration of carboxylate RCOO– [21]. Another new peak at 1725 cm^−1^ was observed in OSA-modified starches, which can be attributed to the characteristic C=O stretching vibration of an ester carbonyl group [27]. These results confirmed that native normal and high-amylose starches were successfully esterified by OSA. OSA-HighAmyST-2 showed higher peak heights at 1571 and 1725 cm^−1^ than OSA-HighAmyST-1. This result suggests that the increase of DS enhances the ester band absorption of OSA-modified high-amylose Japonica starch.

### 3.3. Morphologies and Particle Size Distributions of OSA-Modified Japonica Rice Starches

**Figure 3:**
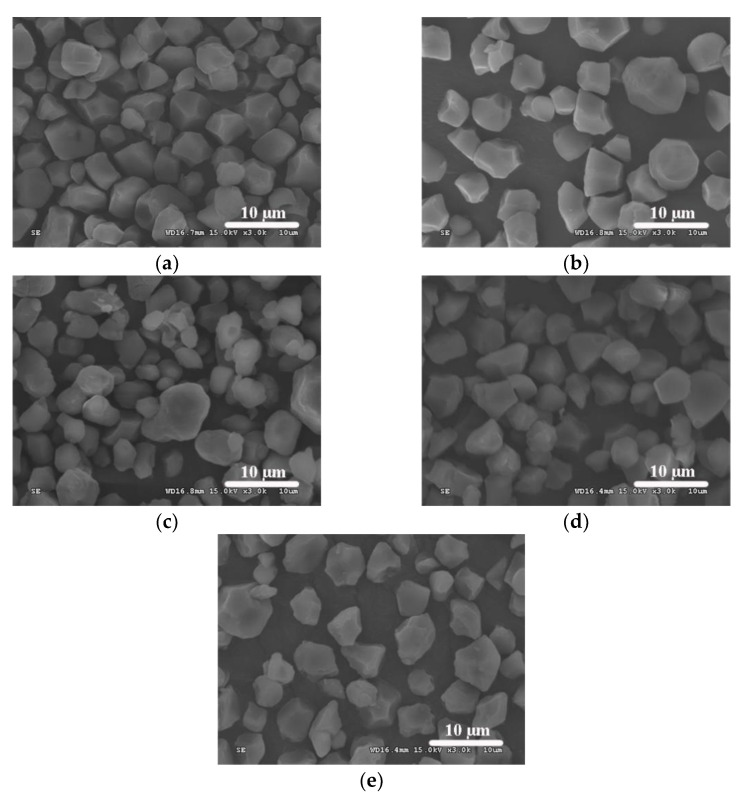
Scanning electron microscopy (SEM) images of normal and high-amylose Japonica rice starches with and without OSA modification. (**a**) NormalST: native normal starch; (**b**) OSA-NormalST: OSA-modified normal starch (DS = 0.0283); (**c**) HighAmyST: native high-amylose starch; (**d**) OSA-HighAmyST-1: OSA-modified high-amylose starch (DS = 0.0177); (**e**) OSA-HighAmyST-2: OSA-modified high-amylose starch (DS = 0.0285).

**Figure 4:**
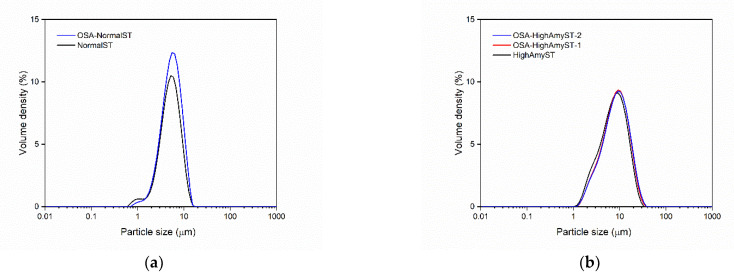
Particle size distribution curves of normal (**a**) and high-amylose Japonica rice starches (**b**) with and without OSA modification. NormalST: native normal starch; OSA-NormalST: OSA-modified normal starch (DS = 0.0283); HighAmyST: native high-amylose starch; OSA-HighAmyST-1: OSA-modified high-amylose starch (DS = 0.0177); OSA-HighAmyST-2: OSA-modified high-amylose starch (DS = 0.0285).

The SEM images of normal and high-amylose Japonica rice starches with and without OSA modification are shown in Figure 3, and their particles size distribution curves are shown in Figure 4. In general, OSA modification did not change the surface morphology of granules for both normal and high-amylose Japonica rice starches. This result was consistent with Bai and Shi [28] in the research of OSA-modified maize starch. For both starch varieties, native starch and OSA-modified starch showed similar particle size distribution curves. The volume-average particle diameter (D[4,3]) of native high-amylose starch was 8.67 μm. The D[4,3] values of OSA-modified high-amylose starches at DS of 0.0177 and 0.0285 were 8.56 and 8.87 μm, respectively. However, there were no significant differences between native high-amylose starch and OSA-modified derivatives in D[4,3] (*p* > 0.05). These results suggest that particle size distribution of high-amylose starch was not significantly influenced by the OSA modification.

### 3.4. Crystalline Structures of OSA-Modified Japonica Rice Starches

**Figure 5:**
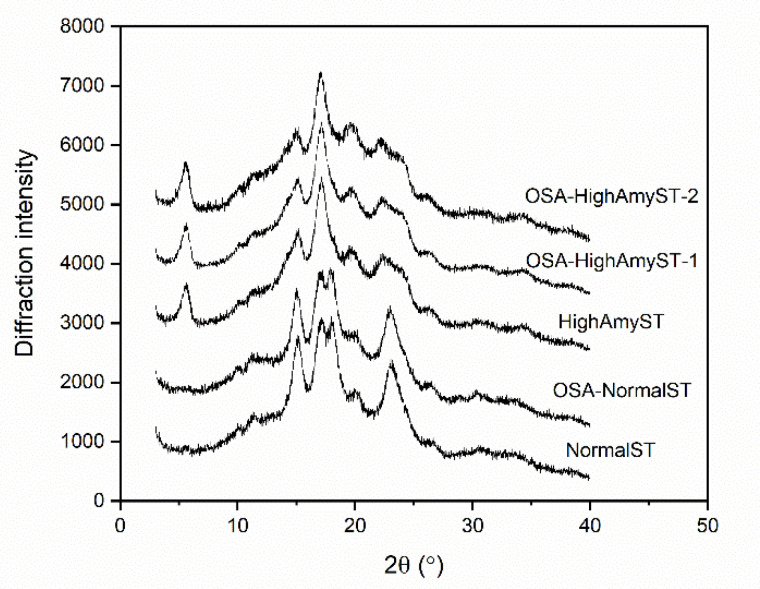
X-ray diffraction (XRD) patterns of normal and high-amylose Japonica rice starches with and without OSA modification. NormalST: native normal starch; OSA-NormalST: OSA-modified normal starch (DS = 0.0283); HighAmyST: native high-amylose starch; OSA-HighAmyST-1: OSA-modified high-amylose starch (DS = 0.0177); OSA-HighAmyST-2: OSA-modified high-amylose starch (DS = 0.0285).

XRD patterns of normal and high-amylose Japonica rice starches with and without OSA modification are shown in Figure 5. Native normal starch showed an A-type crystalline pattern, with characteristic peaks appearing at 2θ of 15.0, 17.0, 17.9, and 23.0°. Native high-amylose starch exhibited characteristic peaks at 2θ of 5.6, 15.0, 17.0, 19.6, and 23.1°, indicating a C-type crystalline pattern. OSA modification did not change the diffraction patterns of normal and high-amylose starches. For both starch varieties, relative crystallinities (RCs) of OSA-modified starches were slightly lower than those of native starches (*p* < 0.05). The RC of native high-amylose starch was 28.3%, and the derivatives at DS of 0.0177 and 0.0285 were significantly lower at 27.5% and 27.1%, respectively. This was consistent with previous research on OSA modification of indica rice starch and corn starch [13,29]. These results suggest that the esterification of OSA mainly occurred at the amorphous regions of high-amylose starch granules and made a slight difference to the crystalline structure.

### 3.5. Pasting Properties of OSA-Modified Japonica Rice Starches

The pasting parameters of normal and high-amylose Japonica rice starches with and without OSA modification are shown in Table 1. Native high-amylose starch had higher PT but lower PV and BDV than native high-amylose starch (*p* < 0.05). This result was consistent with some previous research on other starches [30,31,32]. Zhou et al. [30] reported that the increase of amylose content can strengthen the stability of crystals and delay the gelatinization of starch. Raina et al. [31] reported that low-amylose starches reached peak viscosities at lower temperatures than high-amylose starches and presented higher peak viscosities. Park et al. [32] suggested that starch containing more amylose content and long-length chain amounts of amylopectin were more resistant to swelling, thus showing a lower pasting viscosity. The PT of both normal and high-amylose starches decreased (*p* < 0.05) after OSA modification, which can be attributed to the weakening of the intermolecular hydrogen bond induced by the introduction of the bulky OSA group [33]. Similar results were reported with indica rice starch and other starches [13,34]. For both starch varieties, OSA-modified starch had higher pasting viscosity parameters (PV, TRV, BDV, FV, and SBV) than native starch (*p* < 0.05). For OSA-modified high-amylose starch, pasting viscosities generally changed as DS increased. The incorporation of bulky OSA groups enhanced the overall pasting capacity of the starches, and the modified starches tended to paste more extensively [13]. Different from native starches, OSA-modified high-amylose starch had a higher peak viscosity than OSA-modified normal starch of similar DS. This result suggests that, compared with normal starch, the pasting viscosity of high-amylose starch was more affected by OSA modification. For high-amylose Japonica rice starch, the increase of pasting viscosity induced by OSA modification was beneficial for its application as a thickener in food systems.

### 3.6. Swelling Powers of OSA-Modified Japonica Rice Starches

The swelling powers of normal and high-amylose Japonica rice starches with and without OSA modification are shown in Table 2. As heating temperature increased from 50 to 80 °C, the swelling power of native normal starch increased from 2.00 to 16.17 *g*/*g*, while that for native high-amylose starch increased from 2.13 to 7.47 *g*/*g*. The increase of amylose and long chains of amylopectin can strengthen the granular structure of starch and inhibit the swelling of granules during heating [32,35]. After OSA modification, both normal and high-amylose starches exhibited an increase in swelling power (*p* < 0.05). This may be ascribed to the weakening of the intermolecular hydrogen bond due to the introduction of OSA groups [33]. Like native starches, swelling power (60–80 °C) of OSA-modified high-amylose starch was lower than OSA-modified normal starch of similar DS (*p* < 0.05). The two OSA-modified high-amylose starches with different DS had a slight difference in swelling power.

### 3.7. Emulsifying Stabilities of OSA-Modified Japonica Rice Starches

The creaming indexes of normal and high-amylose Japonica rice starches with and without OSA modification are shown in Table 3. The emulsions of native normal and high-amylose starches did not have a significant difference in creaming index (*p* > 0.05). For normal starch, OSA modification improved the emulsifying stability, as revealed by the decreased creaming indexes of both freshly prepared and stored emulsions (*p* < 0.05). Compared with emulsions of native high-amylose starch, emulsion of OSA-HighAmyST-1 showed no significant difference in the creaming index of the freshly prepared emulsion (*p* > 0.05), but showed a slight decrease in the creaming index of the stored emulsion (*p* < 0.05), suggesting that the emulsifying stability of high-amylose starch was slightly improved after a low degree of OSA modification. The increase of DS enhanced the emulsifying stability of OSA-modified high-amylose starch. For both freshly prepared and stored emulsions, there were no significant differences between the OSA-HighAmyST-2-formulated emulsion and the OSA-NormalST-formulated emulsion in creaming indexes (*p* > 0.05). This result suggests OSA-modified high-amylose starch had an emulsifying stability comparable to OSA-modified normal starch of similar DS.

### 3.8. In Vitro Digestiblities of OSA-Modified Japonica Rice Starches

The RDS, SDS, and RS contents of normal and high-amylose Japonica rice starches with and without OSA modification are shown in Table 4. As compared to normal rice starches, high-amylose starch contained higher RS content and lower RDS content (*p* < 0.05). This result could be attributed to its higher amylose content and higher amounts of long-length chains (B2 and B3 chains). You et al. [36] suggested that RS content is positively correlated with amylose content and long chain amounts of amylopectin. For both starch varieties, the OSA modification increased RS content and decreased the RDS content (*p* < 0.05). RS of OSA-modified high-amylose starch increased as DS increased (*p* < 0.05). Similar results were obtained by Remya et al. [37], who reported that OSA substitution has a positive effect in the formation of RS fraction in potato and cassava starches. At similar DS, RS of OSA-modified high-amylose starch was higher than that of OSA-modified normal starch (*p* < 0.05). This result was in agreement with the difference of their native starches in RS content.

## 4. Conclusions

OSA-modified high-amylose Japonica rice starch with different DS were prepared. The FTIR spectra confirmed the formation of ester linkage between OSA and starch. Compared with native high-amylose starch, the derivative did not show a significant change in morphology and particle size. OSA modification slightly decreased the relative crystallinity and pasting temperature of high-amylose starch, but increased the pasting viscosity, swelling power, emulsifying stability, and RS content. The changes of these properties were affected by DS. Compared with OSA-modified normal starch of similar DS, the OSA-modified high-amylose starch had a higher peak viscosity and RS content, lower swelling power, and comparable emulsifying stability. The modified high-amylose Japonica rice starch could potentially be employed as emulsifier, thickener, and fat replacer in the food industry, and the application needs further study. Due to the high content of RS, this starch derivative may have many potential health benefits, such as decreasing postprandial glycemic and insulinemic responses, lowering plasma triglyceride concentrations, improving whole-body insulin sensitivity, and reducing fat storage, which also deserve further research attention.

## Figures and Tables

**Table 1:** Pasting parameters of normal and high-amylose Japonica rice starches with and without OSA modification ^†^.

	PT (°C)	Ptime (min)	PV (cp)	TRV (cp)	BDV (cp)	FV (cp)	SBV (cp)
Normal-ST	74.4 ± 0.7 ^d^	4.35 ± 0.02 ^c^	3220 ± 45 ^b^	1146 ± 71 ^b^	2074 ± 111 ^b^	2242 ± 126 ^b^	1095 ± 68 ^b^
OSA-NormalST	64.4 ± 0.1 ^a^	3.66 ± 0.01 ^a^	5516 ± 123 ^d^	3014 ± 77 ^e^	2502 ± 46 ^c^	5288 ± 144 ^e^	2274 ± 79 ^c^
HighAmyST	85.8 ± 0.8 ^e^	5.40 ± 0.03 ^e^	1275 ± 21 ^a^	1052 ± 7 ^a^	223 ± 22 ^a^	1785 ± 64 ^a^	733 ± 58 ^a^
OSA-HighAmyST-1	67.5 ± 0.5 ^b^	3.83 ± 0.04 ^b^	5373 ± 93 ^c^	1995 ± 47 ^c^	3378 ± 51 ^d^	4547 ± 36 ^c^	2552 ± 14 ^e^
OSA-HighAmyST-2	69.7 ± 1.7 ^c^	4.44 ± 0.08 ^d^	5730 ± 27 ^e^	2367 ± 25 ^d^	3363 ± 33 ^d^	4784 ± 16 ^d^	2418 ± 35 ^d^

^†^ Data are expressed as means ± SD. Means within a column that had the same letter were not significantly different (α = 0.05). NormalST: native normal starch; OSA-NormalST: OSA-modified normal starch (DS = 0.0283); HighAmyST: native high-amylose starch; OSA-HighAmyST-1: OSA-modified high-amylose starch (DS = 0.0177); OSA-HighAmyST-2: OSA-modified high-amylose starch (DS = 0.0285); PT: pasting temperature; Ptime: peak viscosity time; PV: peak viscosity, TRV: trough viscosity; BDV: breakdown viscosity; FV: final viscosity; SBV: setback viscosity.

**Table 2:** Swelling powers of normal and high-amylose Japonica rice starches with and without OSA modification ^†^.

	Swelling Powers (*g*/*g*)			
	50 °C	60 °C	70 °C	80 °C
NormalST	2.00 ± 0.04 ^a^	2.68 ± 0.01 ^a^	11.54 ± 0.48 ^b^	16.17 ± 0.09 ^b^
OSA-NormalST	6.42 ± 0.23 ^c^	17.28 ± 0.30 ^d^	22.33 ± 0.07 ^e^	23.47 ± 0.48 ^d^
HighAmyST	2.13 ± 0.04 ^a^	2.31 ± 0.01 ^a^	4.32 ± 0.06 ^a^	7.47 ± 0.40 ^a^
OSA-HighAmyST-1	4.40 ± 0.01 ^b^	11.29 ± 0.16 ^c^	17.24 ± 0.05 ^c^	22.32 ± 0.36 ^c^
OSA-HighAmyST-2	6.23 ± 0.23 ^c^	10.54 ± 0.45 ^b^	18.42 ± 0.19 ^d^	22.08 ± 0.31 ^c^

^†^ Data ware expressed as means ± SD. Means within a column that had the same letter were not significantly different (α = 0.05). NormalST: native normal starch; OSA-NormalST: OSA-modified normal starch (DS = 0.0283); HighAmyST: native high-amylose starch; OSA-HighAmyST-1: OSA-modified high-amylose starch (DS = 0.0177); OSA-HighAmyST-2: OSA-modified high-amylose starch (DS = 0.0285).

**Table 3:** Creaming indexes of emulsions prepared from normal and high-amylose Japonica rice starches with and without OSA modification ^†^.

	Creaming Index (%) of Emulsion	
	Freshly Prepared Emulsion	Emulsion Stored for 48 h
NormalST	34.1 ± 2.9 ^b^	38.9 ± 1.0 ^c^
OSA-NormalST	24.3 ± 1.0 ^a^	28.9 ± 0.5 ^a^
HighAmyST	34.5 ± 1.7 ^b^	37.9 ± 1.7 ^c^
OSA-HighAmyST-1	34.2 ± 2.0 ^b^	35.2 ± 0.3 ^b^
OSA-HighAmyST-2	27.9 ± 2.4 ^a^	30.3 ± 0.7 ^a^

^†^ Data are expressed as means ± SD. Means within a column that had the same letter were not significantly different (α = 0.05). NormalST: native normal starch; OSA-NormalST: OSA-modified normal starch (DS = 0.0283); HighAmyST: native high-amylose starch; OSA-HighAmyST-1: OSA-modified high-amylose starch (DS = 0.0177); OSA-HighAmyST-2: OSA-modified high-amylose starch (DS = 0.0285).

**Table 4:** RDS, SDS, and RS contents of normal and high-amylose Japonica rice starches with and without OSA modification ^†^.

	RDS (%)	SDS (%)	RS (%)
NormalST	84.04 ± 0.42 ^e^	11.09 ± 0.17 ^b^	4.87 ± 0.24 ^a^
OSA-NormalST	67.64 ± 1.77 ^b^	7.05 ± 1.08 ^a^	25.30 ± 0.69 ^d^
HighAmyST	78.74 ± 0.17 ^d^	9.56 ± 0.37 ^b^	11.70 ± 0.42 ^b^
OSA-HighAmyST-1	71.32 ± 1.15 ^c^	6.59 ± 1.51 ^a^	22.08 ± 0.36 ^c^
OSA-HighAmyST-2	53.24 ± 0.05 ^a^	11.31 ± 0.36 ^b^	35.45 ± 0.42 ^e^

^†^ Data are expressed as means ± SD. Means within a column that had the same letter were not significantly different (α = 0.05). NormalST: native normal starch; OSA-NormalST: OSA-modified normal starch (DS = 0.0283); HighAmyST: native high-amylose starch; OSA-HighAmyST-1: OSA-modified high-amylose starch (DS = 0.0177); OSA-HighAmyST-2: OSA-modified high-amylose starch (DS = 0.0285).

## Data Availability

The data presented in this study are available on request from the corresponding author.
